# Delayed Postoperative Spinal Epidural Hematoma After Thoracic OPLL Surgery: A Case Report and Literature Review

**DOI:** 10.1002/ccr3.71810

**Published:** 2026-01-04

**Authors:** Hayato Takei, Tetsuro Ohba, Nobuki Tanaka, Kotaro Oda, Kai Mizukami, Go Goto, Hirotaka Haro

**Affiliations:** ^1^ Department of Orthopaedic Surgery University of Yamanashi Yamanashi Japan

**Keywords:** case report, decompression, delayed complications, delayed spinal epidural hematomafactor XIII deficiency, spinal epidural hematoma, spine surgery, thoracic OPLL, thoracic ossification of the posterior longitudinal ligament

## Abstract

New axial pain with rapid neurological decline weeks after surgery for thoracic ossification of the posterior longitudinal ligament may signal delayed postoperative epidural hematoma. Immediate MRI and urgent decompression are critical. Consider occult clot‐stabilization defects such as factor XIII deficiency when routine coagulation tests are normal.

## 1 Introduction

Postoperative spinal epidural hematoma (PSEH) is an uncommon but potentially catastrophic complication of spine surgery, with symptomatic cases requiring surgical evacuation estimated at roughly 0.3%–0.5% across procedures [1, 2, 3]. Most PSEH presents early—within 24–72 h after surgery—when vigilance is highest [1, 2]. By contrast, delayed PSEH (onset > 3 postoperative days) is rare (≈0.16% of cases) and therefore easily overlooked as recovery progresses [1, 2, 3].

Thoracic ossification of the posterior longitudinal ligament (OPLL) is recognized as a high‐risk context for perioperative complications and postoperative neurological deterioration compared with other spinal regions [4, 5]. Large decompressions spanning multiple thoracic levels can expose a broad epidural venous plexus and create conditions in which even a small‐volume hematoma can cause severe cord compression. Although delayed PSEH within 10–14 days has been reported [4], presentations beyond two weeks are rare [6, 7, 8, 9, 10, 11, 12].

We describe an unusually late case of DPSEH occurring on POD 25 after thoracic OPLL decompression and fusion. We synthesize current evidence on timing, risk factors, and pathophysiology—including the role of blood‐pressure surges, obesity, multilevel surgery, and occult clot‐stabilization defects such as factor XIII deficiency—to inform recognition and management [2, 3, 13, 14, 15, 16, 17, 18, 19, 20].

## 2 Case History/Examination

A 40‐year‐old obese man (body mass index 32) with progressive thoracic myelopathy presented three years after a C2–C7 posterior decompression and fusion for cervical OPLL. Comorbidities: obesity. No history of coagulopathy or anticoagulant/antiplatelet use. Baseline neurological status included spastic gait, lower‐extremity hyperreflexia, ankle clonus, and impaired vibration sense below mid‐thoracic levels; motor power ≥ 4/5 (Frankel D).

**FIGURE 1:**
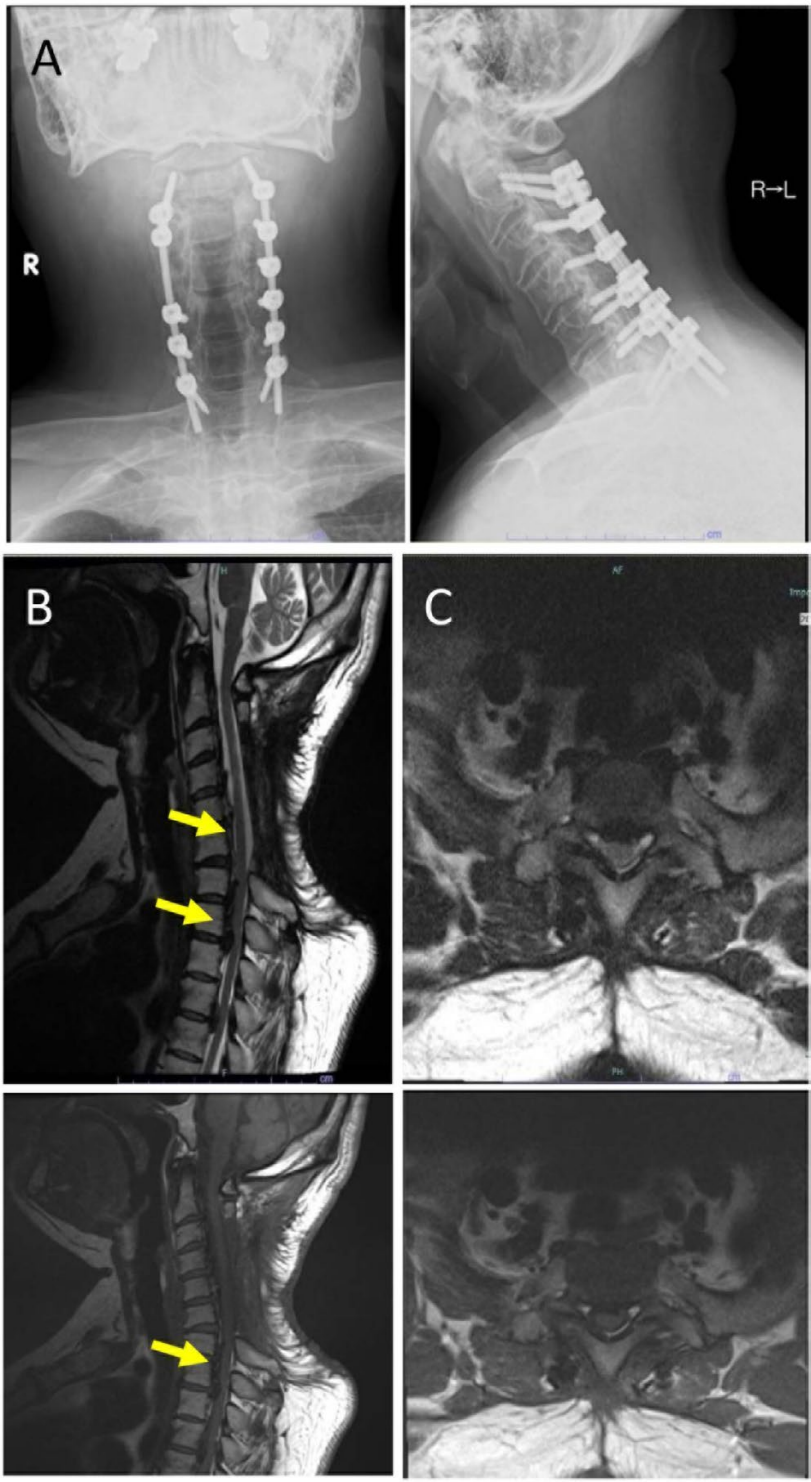
Preoperative radiological evaluation. (A) Preoperative radiographs for the revision procedure. (B) Sagittal T2‐weighted image shows severe dorsal cord compression from T1 to T7 due to OPLL. (C) Axial T1‐ and T2‐weighted image at T2 demonstrates near‐circumferential canal compromise with ventral and dorsal indentation.

Laboratory tests were within normal limits. Preoperative MRI demonstrated severe dorsal compression at mid–upper thoracic levels due to contiguous/segmental OPLL (Figure 1A,B). Standing radiographs showed significant deformity. Anesthetic assessment rated the patient as ASA class 2.

**FIGURE 2:**
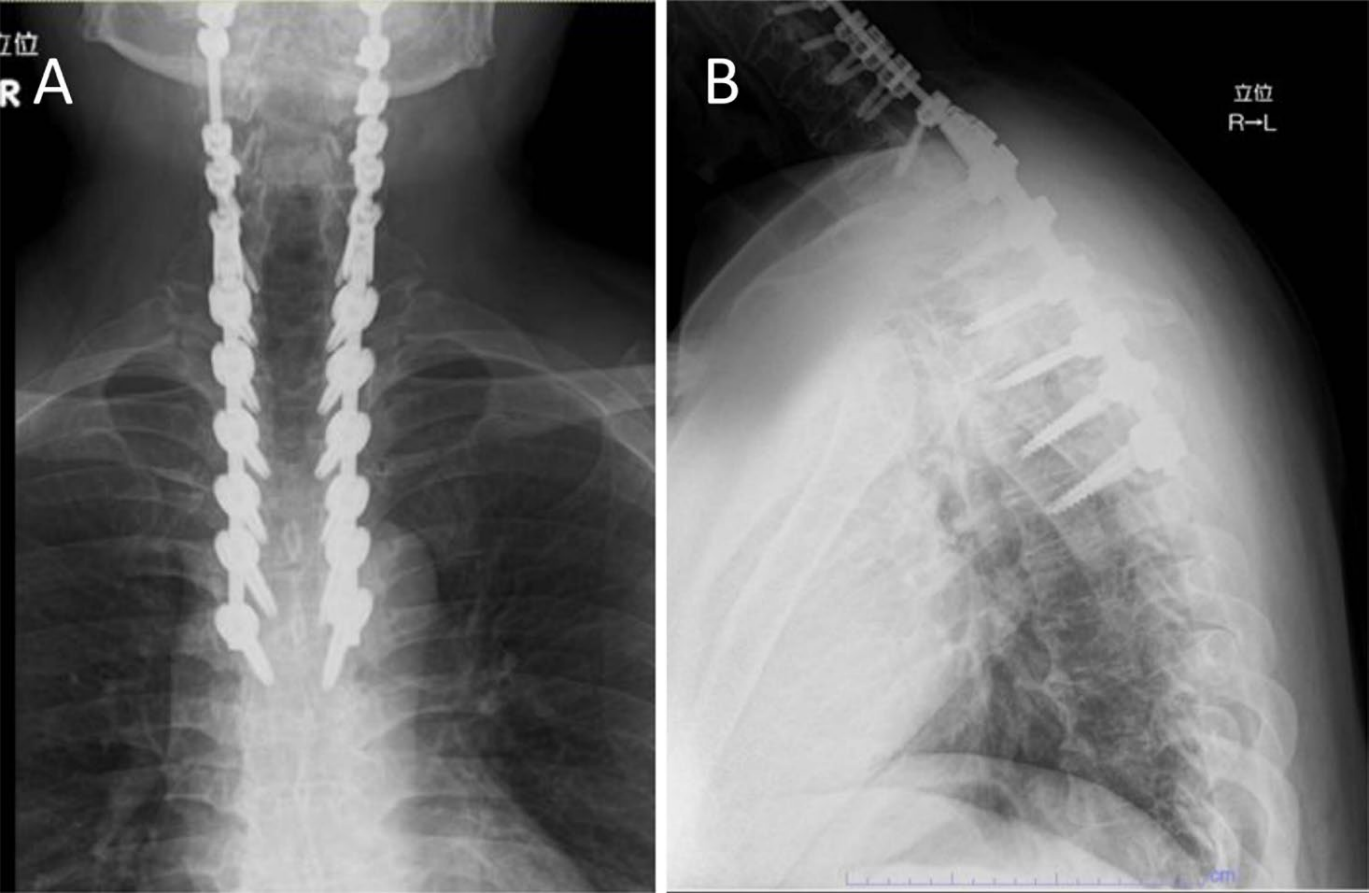
Postoperative radiographs after thoracic decompression and fusion. (A) AP view shows cephalad extension of the pedicle‐screw/rod construct with satisfactory coronal alignment. (B) Lateral view confirms stable sagittal alignment without implant failure.

T2–T7 posterior decompression and instrumented fusion were performed to extend the prior construct (Figure 2). Approach: midline posterior; laminectomies T2–T7; removal/reduction of ossified ligament where needed; pedicle screws T2–T7 connected to the existing C2–C7 construct. Operative duration 5 h; estimated blood loss 630 mL. Intraoperative measures included meticulous hemostasis (bipolar cautery, hemostatic matrix) and placement of a subfascial closed‐suction drain at closure. Mean arterial pressure was maintained at 75–85 mmHg. No neuromonitoring alerts occurred.

Drain output was modest, and the drain was removed on POD 2. The patient mobilized with a thoracolumbar orthosis beginning POD 3–5 and participated in supervised gait training. Neurological status remained stable relative to baseline. On POD 10 he was transferred to an inpatient rehabilitation facility with instructions to avoid excessive strain and to maintain blood pressure control at 100–120 mmHg.

**FIGURE 3:**
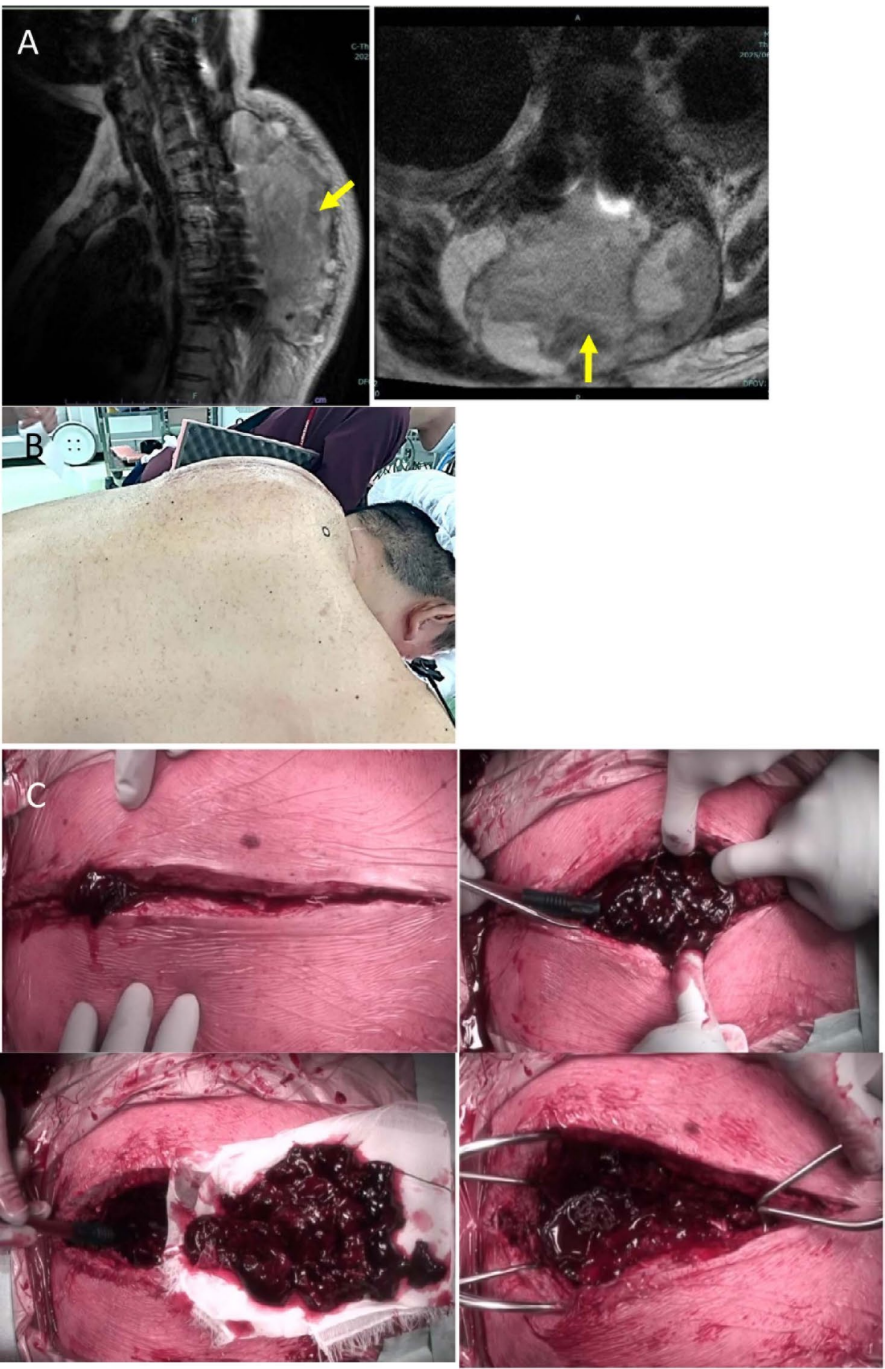
Images of emergent evacuation of postoperative epidural hematoma (POD 25). (A) Sagittal and axial T2‐weighted MRI reveal a large dorsal epidural mass from T4 to T9 producing marked cord compression. (B) Intraoperative photograph showing marked dorsal soft tissue swelling. (C) Pre‐ and post‐evacuation images confirm a massive hematoma and its removal.

During routine rehabilitation on POD 25, the patient developed sudden severe interscapular pain followed within 1–2 h by rapidly progressive bilateral leg weakness (Frankel B), a T5 sensory level, and urinary retention. He denied any fall or direct trauma. According to the rehabilitation report, he was in a supine position performing only mild stretching when the back pain started; thus, excessive physical load was unlikely to have triggered the event. No marked blood‐pressure surges were documented around symptom onset. Emergent MRI demonstrated a large dorsal epidural collection extending from approximately T4 to T9, causing marked spinal cord compression (Figure 3A).

## 3 Differential Diagnosis, Investigations and Treatment

In cases of acute neurological decline in the late postoperative period, differential diagnoses include epidural hematoma, spinal epidural abscess, seroma or cerebrospinal fluid collection, and hardware failure or progression of underlying spinal pathology. In this patient, the absence of any fall or trauma and lack of systemic infection signs made a compressive hematoma the most likely cause, which was promptly confirmed by MRI.

The prior incision was reopened urgently. A tense dorsal epidural hematoma was encountered, and approximately 500 mL of clotted blood was evacuated; this volume was estimated from suction canister volume and surgical sponges (Figure 3B,C). No active arterial bleeder was identified; the bleeding pattern suggested a venous source. Hemostasis was achieved and new drains were placed.

Standard coagulation parameters (PT/INR, aPTT, platelet count, fibrinogen) were normal. Extended evaluation showed plasma factor XIII activity approximately 50% of normal (Table 1), suggestive of partial deficiency. Postoperatively, cryoprecipitate was administered for temporary factor XIII replacement and strict blood pressure control was instituted (systolic < 140 mmHg). Further testing (von Willebrand panel, factor VIII/IX) was unremarkable.

**TABLE 1:** Postoperative coagulation and fibrinolysis laboratory results.

Test (units)	Result	Reference range
PT‐INR	1.11	0.9–1.1
APTT (seconds)	29.3	25–35
Fibrinogen (mg/dL)	333	200–400
D‐dimer (μg/mL)	1.4 ↑	< 1.0
Factor XIII (%)	57 ↓	70–140
Factor XIII: Ag (%)	50 ↓	70–140

Abbreviations: PT/INR = prothrombin time–international normalized ratio; aPTT = activated partial thromboplastin time; Ag = antigen. ↑ above normal; ↓ below normal.

## 4 Conclusion and Results (Outcome and Follow‐Up)

**FIGURE 4:**
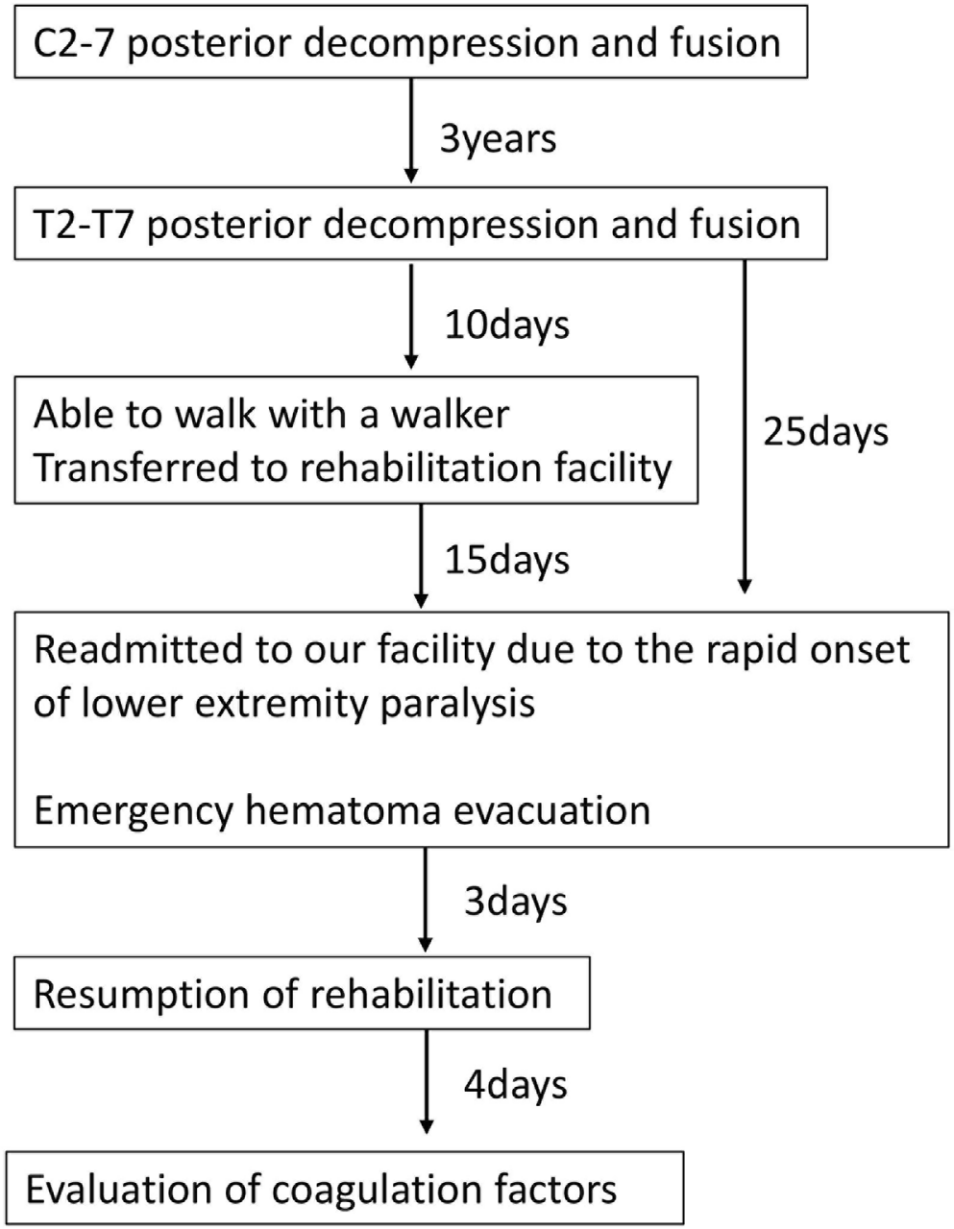
Timeline of the clinical course. Three years after C2–7 posterior decompression and fusion, the patient underwent T2–T7 posterior decompression and fusion. Ten days after the thoracic surgery, the patient was able to ambulate with a walker and was transferred to a rehabilitation facility. Fifteen days later (25 days after the thoracic surgery), the patient was readmitted to the hospital due to rapid‐onset lower‐extremity paralysis and underwent emergency hematoma evacuation. Rehabilitation was resumed 3 days after hematoma evacuation, and coagulation factors were evaluated 4 days thereafter. *Numbers indicate the time interval between events*.

After 48 h of flat bed rest following hematoma evacuation, gradual remobilization was initiated. Motor strength improved to 4–5/5 by two weeks after evacuation (Frankel D); at three months post‐op he ambulated with a walker (Frankel D). Bladder function recovered, with only minimal residual nocturnal incontinence. A summary timeline of key events is provided in Figure 4.

DPSEH can complicate thoracic OPLL surgery even several weeks after an apparently uneventful recovery. Clinicians should maintain a high index of suspicion for hematoma whenever new pain or neurological decline emerges at any postoperative time point. Rapid MRI and urgent decompression are pivotal for neurological salvage. In atypical late presentations without trauma or anticoagulants and with normal routine coagulation studies, selective testing for occult clot‐stabilization defects (e.g., factor XIII deficiency) may help guide perioperative management and reduce recurrence risk.

## 5 Discussion

Most symptomatic PSEH manifests within 24–72 h after spine surgery [1, 2, 3]. Among delayed cases (> 3 days), onset peaks around POD 3–8 and declines thereafter; very late presentations (≥POD 14) are rare but documented (Table 1). Our POD‐25 case after thoracic OPLL surgery, to our knowledge, is among the latest reported cases after thoracic OPLL surgery and underscores the need for prolonged postoperative vigilance [12]. In addition, we conducted a targeted PubMed literature search using keywords such as “postoperative spinal epidural hematoma,” “delayed,” “thoracic,” and “OPLL,” and identified only a small number of reports with symptom onset ≥ 10 days after surgery (Table 2).

**TABLE 2:** Previously reported cases of delayed postoperative spinal epidural hematoma (onset ≥ 10 days after surgery). Note: Cases of symptomatic PSEH with onset ≥ 10 days after surgery identified in the literature search.

Source (year)	Patient	Initial Surgery (level)	Hematoma Onset	Presentation	Treatment	Outcome
Spanier et al. (2000) [11]	68‐year‐old male	Lumbar laminectomy (L4–L5); post‐op heparin	POD 16	Acute cauda equina	Urgent laminectomy and evacuation	Marked improvement
Caruso et al. (2013) [12]	65‐year‐old male	C6 corpectomy with fusion	POD 20 and POD 37	Neck pain, quadriplegia; recurrent paralysis	Two surgical evacuations	Partial recovery
Morooka et al. (2024) [10]	41‐year‐old male	T2–T3 laminectomy; T1–T4 fusion (OPLL)	POD 12	Upper back pain, paraplegia	Emergent evacuation (T1–T4)	Gradual improvement

Abbreviations: OPLL = ossification of the posterior longitudinal ligament; POD = postoperative day.

Multiple patient and surgical factors contribute to PSEH risk. Reported associations include multilevel procedures, prior surgery at the same site, obesity, perioperative hypertension or acute postoperative blood‐pressure surges, substantial blood loss, anemia, and coagulopathy [13, 15, 16]. In particular, a systolic surge ≥ 50 mmHg at extubation and higher body mass index have been identified as independent risks [14]. Thoracic procedures—and thoracic OPLL in particular—are associated with higher complication and neurological deterioration rates than cervical or lumbar procedures [4, 5].

Early PSEH likely reflects inadequate hemostasis, persistent epidural venous oozing, drain malfunction/occlusion, and abrupt hemodynamic changes. Delayed PSEH may involve gradual accumulation from low‐pressure venous sources within a large decompression bed, activity‐related Valsalva stresses during rehabilitation, and late clot destabilization. Notably, drains do not eliminate risk and hematomas can accumulate despite drainage [1, 2].

Routine PT/aPTT and platelet counts may be normal in patients with clot‐stabilization defects. Factor XIII deficiency impairs fibrin cross‐linking, predisposing to delayed rebleeding despite apparently normal coagulation screens [18]. Case literature links factor XIII deficiency to spontaneous epidural hemorrhage [19], highlighting that severe bleeding can occur despite normal standard coagulation tests. Our patient's subnormal factor XIII activity (~50%) may have contributed to delayed hematoma formation. However, factor XIII activity was measured only once postoperatively; therefore, it remains unclear whether this represented a mild congenital deficiency or an acquired reduction related to surgery/consumption. No repeat factor XIII testing, genetic analysis, or family studies were performed, which we acknowledge as a limitation. In similar cases, hematology consultation is advisable, and if clinically significant factor XIII deficiency is confirmed, perioperative factor XIII supplementation (e.g., cryoprecipitate or factor XIII concentrate) may be considered in future surgeries to reduce bleeding risk.

Any new axial pain combined with rapid neurological change at any postoperative time should prompt urgent MRI to exclude PSEH. Neurologic outcome correlates strongly with time to decompression; expedited evacuation offers the best chance of recovery [17]. Preventive strategies include meticulous intraoperative hemostasis, adequate drainage with vigilance for malfunction, careful blood‐pressure control, graduated rehabilitation avoiding early Valsalva stresses, and targeted hematology consultation when bleeding is unexplained by routine tests. Finally, this single‐case report has limitations including limited generalizability, incomplete characterization of the factor XIII abnormality, and relatively short follow‐up.

## Author Contributions

**Hayato Takei:** conceptualization, formal analysis, funding acquisition, investigation, methodology, validation, writing – original draft. **Tetsuro Ohba:** conceptualization, data curation, formal analysis, supervision, writing – original draft, writing – review and editing. **Nobuki Tanaka:** data curation, formal analysis, methodology. **Kotaro Oda:** resources, validation, visualization. **Kai Mizukami:** data curation, project administration. **Go Goto:** methodology, visualization. **Hirotaka Haro:** supervision, validation, visualization.

## Funding

No specific funding was received for this work.

## Ethics Statement

The patient provided informed consent for publication of this case report and associated images. The report complies with institutional and journal ethical standards. Written informed consent has been obtained.

## Consent

Written informed consent for publication (text and images) was obtained from the patient.

## Conflicts of Interest

The authors declare no conflicts of interest related to this work.

## Data Availability

The data that support the findings of this study are available on request from the corresponding author. The data are not publicly available due to privacy or ethical restrictions.
